# New Appliances and Things Medical

**Published:** 1894-09-08

**Authors:** 


### NEW APPLIANCES AND THINGS MEDICAL.

[All preparations, appliances, novelties, &c., of which a notice is desired, should be sent for the Editor, to care of The Manager 428, Strand, London, W.C.]

### THE "TELL-TALE" MILK JUG.

J. Lawrence, 56, Fulham Road, S.W., sends us a pint specimen in glass of the above. There are other two sizes, one quart and two quarts. Marked on the side of the jug are divisions showing a pint, one-half, three-quarters, and one-quarter of a pint. These divisions enable a customer on taking in the milk to see at a glance that full measure has been given. Besides these marks there are others, viz., "average," "good," and "very good;" these are arranged to show the percentage of cream present, if that is desired. The "Tell-tale" milk jug embodies an ingenious and simple idea, the price is most reasonable, and those who once use this jug are not likely to be again contented with any other.

### NATURAL MINERAL WATER FROM HAARLEM, HOLLAND.

(W. Schacht and Co., 26, Finsbury Pavement, E.C.

The introduction of these waters to the British public is the outcome of a praiseworthy attempt on the part of the Dutch to open up in Holland a watering-place possessed of natural springs, such as are possessed by Spa, Schwalbach, St. Moritz, and other watering-places in the different countries of Europe. There can be no doubt that of all chalybeate waters there are few that can compare with that of the Wilhelmina spring at Haarlem, either as regards the quantity of iron contained, or as regards the variety and percentage of the subsidiary constituents. Analysis shows that in 1,000 parts of water iron carbonate can be detected to the extent of 0·1112 parts, and chloride of soda to the extent of 3·24 parts, while representative salts of manganese, magnesia, sodium, potassium, lithium, calcium, ammonia, aluminum in the form of bicarbonate, chloride, sulphate, iodide, bromide, or phosphate are also present in useful proportions. The water as bottled and forwarded to England is slightly aerated, and presents a fine sparkling appearance when poured into the tumbler. Chemical analysis proves that the constituents of this water are such as the therapeutist would combine artificially and prescribe in cases of anæmia or chlorosis, and the practical results which have been already acheived by the Wilhelmina cure, either in the case of patients on the spot or at a distance is highly encouraging and promises well for the future.

